# Retrospective analysis of Vitek^®^2 performance compared to manual broth micro-dilution for colistin susceptibility testing of *Acinetobacter baumannii*complex isolates in South Africa

**DOI:** 10.4102/ajlm.v11i1.1597

**Published:** 2022-02-28

**Authors:** Vuyolwethu Fadana, Teena Thomas, Nina von Knorring

**Affiliations:** 1 School of Pathology, Faculty of Health Sciences, University of the Witwatersrand, Johannesburg, South Africa; 2 Department Pathology, National Health Laboratory Services, Johannesburg, South Africa; 3 Infectious Control Services Laboratory, National Health Laboratory Services, Johannesburg, South Africa; 4 Mycobacteriology Referral Laboratory, National Health Laboratory Service, Johannesburg, South Africa

**Keywords:** Acinetobacter, colistin, broth micro-dilution, Vitek^®^2, antimicrobial susceptibility testing

## Abstract

The manual broth micro-dilution (mBMD) is the recommended reference method for colistin minimum inhibitory concentration determination; however, it is not as readily available in South Africa as the Vitek^®^2. This retrospective study compared the performance of Vitek^®^2 against mBMD in determining the colistin minimum inhibitory concentration of 337 extensively drug-resistant *Acinetobacter baumannii* complex isolates. Vitek^®^2 yielded a categorical agreement of 89%, an essential agreement of 56%, a major error rate of 8% and a very major error rate of 55%. The Vitek^®^2 is not an alternative to mBMD for colistin susceptibility testing.

## Introduction

The increasing antimicrobial resistance in the *Acinetobacter baumannii* complex, and the recently observed resistance to the polymyxin antibiotic (colistin), demands timeous identification and antimicrobial susceptibility profiling of causal pathogens to ensure timely and appropriate antimicrobial therapy decisions.^1,2^ Different laboratory methods for the assessment of colistin susceptibility testing among *A. baumannii* isolates have been assessed.^3^

Since 2016, the reference colistin susceptibility testing method recommended by the Clinical and Laboratory Standards Institute and the European Committee on Antimicrobial Susceptibility Testing is the International Organization for Standardization (ISO) broth micro-dilution (BMD) method (ISO-20776).^4^ Implementing manual BMD (mBMD) is currently not feasible in the routine microbiology laboratory due to its laborious nature.^5^ The Vitek^®^2 (BioMerieux Inc., Marcy l’Étoile, France) is an automated microbial identification and antimicrobial susceptibility testing (AST) platform. Most public and private South African laboratories use the Vitek^®^2, making it an attractive alternative to the mBMD. However, studies comparing Vitek^®^2 and the mBMD method have reported discordant colistin susceptibility results.

Dafopoulou et al. compared the mBMD with polysorbate-80, Vitek^®^2, Etest, agar dilution, and the minimum inhibitory concentration (MIC) methods for the colistin susceptibility testing of 51 *Klebsiella pneumoniae* and 20 *A. baumannii* clinical isolates.^6^ Eighteen of the *A. baumannii* isolates were colistin-resistant by mBMD, and Vitek^®^2 categorically agreed in 85% and essentially agreed in 90% of the *A. baumannii* isolates. There were no ‘very major errors’ (VME) reported. These findings were similar to those obtained by Lo-Ten-Foe et al.^7^ Piewngam and Kiratisin also observed a low VME rate of 0.7% when testing 290 *A. baumannii* isolates.^8^ These findings suggest that Vitek^®^2 could be a viable alternative to the mBMD. In contrast to these results, Vourli et al. reported unacceptably high VME rates for Phoenix100 and Vitek^®^2 against the mBMD (41.4% and 37.9%).^9^ Additionally, in 2017 BioMerieux retracted the use of Vitek^®^2 for colistin testing owing to the Clinical and Laboratory Standards Institute-European Committee on Antimicrobial Susceptibility Testing recommendations and an in-house observed performance issue: high VME rate against agar dilution and mBMD methods.^10^ Due to these contradictory findings in the literature, further research into this area was warranted.

This study aimed to compare the performance of Vitek^®^2 colistin susceptibility testing to mBMD for clinical extensively drug-resistant (XDR) *A. baumannii* complex isolates at Charlotte Maxeke Johannesburg Academic Hospital in Johannesburg, South Africa.

## Methods

### Ethical considerations

Ethical approval was obtained from the University of the Witwatersrand Human Research Ethics Committee (clearance certificate number M191048 MED 19-10-043). Data were anonymised before analysis to maintain patient confidentiality. Patient consent was not required. Approval to utilise patient data was obtained from the Chief Executive Officer of Charlotte Maxeke Johannesburg Academic Hospital.

### Data collection

This was a descriptive, retrospective analysis of the *A. baumannii* complex isolated from Charlotte Maxeke Johannesburg Academic Hospital inpatients between 01 January 2017 and 30 June 2019. The XDR *A. baumannii* complex isolates were resistant to one or more agents in all but one or two categories of antibiotics. Microbiological data, including the sample type, Vitek^®^2 and mBMD colistin MIC results, were extracted from the Corporate Data Warehouse, a division of the National Health Laboratory Service (NHLS).

### Isolate identification and AST

Microbiology services within the hospital are provided by the NHLS. The identification of isolates within the institution was performed using either the Vitek^®^2 (BioMerieux Inc., Marcy l’Étoile, France) Gram-negative Identification (GN ID) card or matrix-assisted laser desorption ionisation-time of flight mass spectrometry. These methods are unable to differentiate species within the *A. baumannii* complex. Routine AST was performed using the Kirby-Bauer disc diffusion susceptibility method or Vitek^®^2 AST-N256 card. Isolates that had AST by the former method were excluded from the study. Quality control strains and pure cultures of test isolates were used for the Vitek^®^2 AST. Isolates were further tested using mBMD when colistin therapy was considered, that is, for clinically significant XDR *A. baumannii* complex isolates. Manual BMD was performed by trained personnel with appropriate controls according to the ISO-20776 recommendation. Isolates that were colistin-resistant by mBMD were then sent to a reference laboratory for MCR 1-5 testing (data not shown).

### Data analysis

All XDR *A. baumannii* complex isolates cultured between 01 January 2017 and 30 June 2019 were analysed. No patient admission data was available to discriminate between community-acquired and hospital-acquired infections. Isolates that were obtained from outpatient departments or without the ward specified were excluded. Duplicate patient samples, such as blood cultures collected within two weeks and other sample types collected within one month of the initial sample, were excluded. Intravenous central venous catheter tips were not included as they were processed in a separate laboratory using a different automated AST platform. Only isolates with both Vitek^®^2 and mBMD colistin susceptibility results were included. The performance of Vitek^®^2 colistin susceptibility testing was determined by evaluating the categorical and essential agreements and the major and VME rates in comparison to the mBMD colistin susceptibility testing method, according to the Food and Drug Administration (FDA) recommendations.^11^

Microsoft^®^ Excel 2016 (Microsoft Corporation, Redmond, Washington, United States) was used for data analysis. The following equations were employed to assess agreement and error rates:

- Categorical agreement = (number of isolates correctly classified by Vitek^®^2 as either colistin susceptible or resistant in comparison to mBMD ÷ total number of isolates tested) × 100     [Eqn 1]
- Essential agreement = (number of isolates within one doubling dilutions of the mBMD MIC on Vitek^®^2 ÷ total number of isolates tested) × 100     [Eqn 2]
- Major error rate = (number of falsely resistant isolates on Vitek^®^2 ÷ number of susceptible isolates by mBMD) × 100     [Eqn 3]
- Very Major error rate = (number of falsely susceptible isolates by Vitek^®^2 ÷ number of resistant isolates by mBMD) × 100     [Eqn 4]

## Results

**FIGURE 1:**
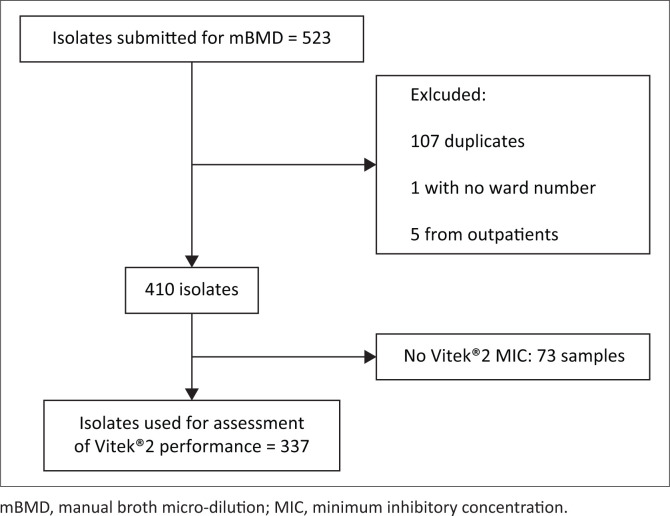
Isolate selection and inclusion for Vitek^®^2 colistin susceptibility assessment.

Data for 523 isolates were obtained from all specimen types that harboured XDR *A. baumannii* complex and were submitted for colistin mBMD. After appropriate exclusions, 337 (64%) isolates had both Vitek^®^2 and mBMD MIC results (Figure 1).

**FIGURE 2:**
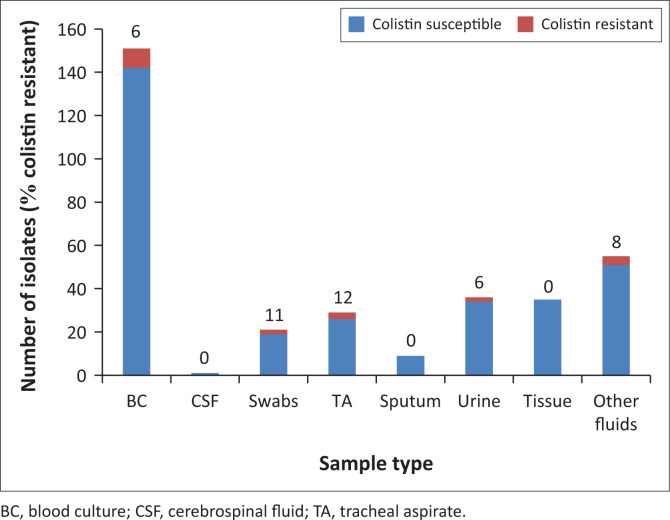
Distribution of colistin-resistant and susceptible XDR *A. baumannii* complex isolates by sample type (*n* = 337).

Of the 337 isolates with both Vitek^®^2 and mBMD colistin susceptibility results, 20 (6%) were resistant to colistin by mBMD. The highest proportion of colistin-resistant *Acinetobacter* was isolated from tracheal aspirates and swabs (Figure 2).

**FIGURE 3:**
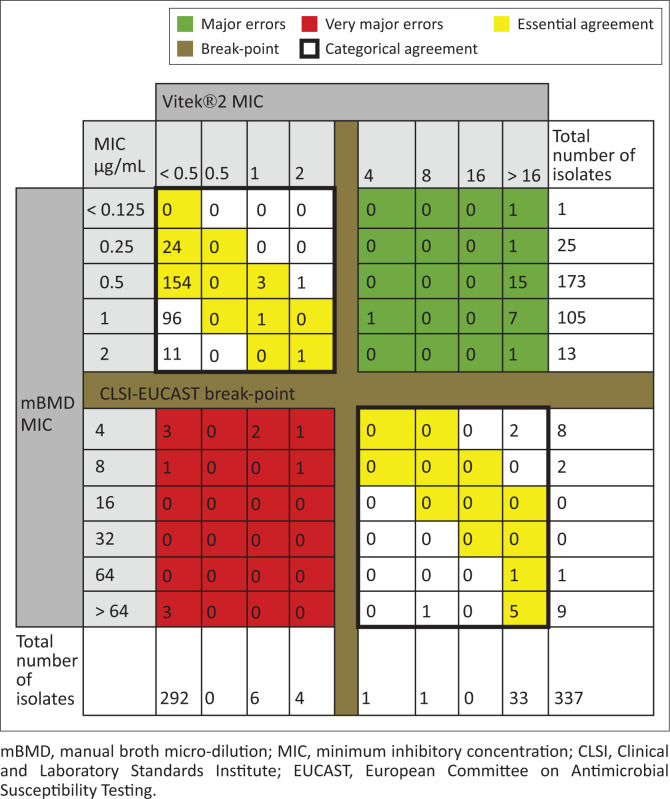
Colistin minimum inhibitory concentration (µg/mL) results for XDR *A. baumannii* complex isolates by both Vitek^®^2 and manual broth micro-dilution.

Vitek^®^2 was found to have a categorical agreement of 89% (300/337) and an essential agreement of 56% (190/337) with mBMD. The VME rate was 55% (11/20) and the major error rate was 8% (26/317) (Figure 3).

## Discussion

Colistin susceptibility testing by mBMD according to ISO-20776 is difficult to implement in routine microbiology laboratories. Due to financial constraints and the technical competencies required, only one NHLS laboratory can offer mBMD in South Africa. This is likely to impair patient care due to delays in turnaround times of results. In contrast, Vitek^®^2 is available in most NHLS microbiology laboratories and serves as an alternative. However, our study demonstrates unacceptable performance, with 11 of 20 (55%) colistin-resistant isolates being falsely susceptible by Vitek^®^2. The Vitek^®^2 also falsely reported some isolates with mBMD colistin MIC of > 64 µg/mL as susceptible. This has the potential to result in inappropriate antimicrobial therapy and adverse patient outcomes. This extremely high VME contrast with previous studies mentioned earlier. However, those studies included fewer *A. baumannii* isolates compared to our study. In addition to the unacceptable VME rate, the categorical agreement, essential agreement and the major error rates with Vitek^®^2 were also unacceptable according to the FDA requirements for an AST testing platform.^11^

Attempts to make mBMD more readily available and easier to implement until other testing options become available are required. Matuschek et al. evaluated five recently developed commercial BMD systems – SEMPA1 (Sensititre™ Custom Plate [Thermo Fisher Scientific, East Grinstead, United Kingdom], MICRONAUT-S and MICRONAUT MIC-Strip [MERLIN Diagnostika GmbH, Bornheim, Germany], SensiTest™ [Liofilchem, Roseto degli Abruzzi, Italy] and UMIC [Biocentric, Bandol, France]).^12^ These were evaluated against mBMD using various gram-negative organisms including 22 *Acinetobacter* spp isolates.^12^ They demonstrated overall better performance compared to our findings with Vitek^®^2. The majority of the platforms had acceptable categorical agreement and essential agreement (> 90%), with significantly lower error rates than obtained in this study. Interestingly, there were no VMEs detected in the *A. baumannii* isolates. However, this study tested fewer isolates than our study. These methods may offer an alternative to mBMD and further research on their performance is required.

### Limitations

Only a small number of colistin-resistant isolates were obtained for the study. Analysis of larger numbers of resistant isolates with wider MIC distribution is required to confirm our finding of high VMEs.

### Conclusion

Based on the results of this study, Vitek^®^2 is not an alternative for mBMD for colistin AST in our setting. Further studies are required to determine if the commercially available colistin BMD methods are a cost-effective option with acceptable analytical performance. Additionally, the semi-automated platforms such as Vitek^®^2 should be better optimised for colistin AST.
